# Tumor Microvasculature: Endothelial Leakiness and Endothelial Pore Size Distribution in a Breast Cancer Model

**DOI:** 10.4137/bcbcr.s481

**Published:** 2008-06-06

**Authors:** E.E. Uzgiris

**Affiliations:** Physics Department, Rensselaer Polytechnic Institute, Troy, NY 12180

**Keywords:** dynamic contrast enhancement MRI, tumor endothelial leakiness, endothelial pore sizes, tumor microvasculature

## Abstract

Tumor endothelial leakiness is quantified in a rat mammary adenocarcinoma model using dynamic contrast enhancement MRI and contrast agents of widely varying sizes. The contrast agents were constructed to be of globular configuration and have their uptake rate into tumor interstitium be driven by the same diffusion process and limited only by the availability of endothelial pores of passable size. It was observed that the endothelial pore distribution has a steep power law dependence on size, r^−^*^β^*, with an exponent of −4.1. The model of large pore dominance in tumor leakiness as reported in some earlier investigation with fluorescent probes and optical chamber methods is rejected for this tumor model and a number of other tumor types including chemically induced tumors. This steep power law dependence on size is also consistent with observations on human breast cancer.

## Introduction

Tumor endothelial leakiness is well known and is associated with the processes of angiogenesis in growing tumors (1). The immature mircovasculature that is associated with angiogenesis has altered hemodynamic parameters: a high fractional vascular volume and permeability to a variety of contrast agents as demonstrated in clinical practice and the use of small molecular weight contrast agents or in preclinical studies with larger molecular weight contrast agents (2–5). The nature of this leakiness has not been well investigated. In particular, how does the permeability vary with probe size? Is the endothelial leakiness dominated by large pores? These questions lead to the more fundamental ones of tumor biology: what is the pore size distribution in the endothelial junctions of tumors? Does the pore size distribution differ between benign and malignant tumors and can such differences be used to better design contrast agent probes for optimum discrimination?

Earlier investigations used fluorescence methods in a tumor models gown in a special, dorsal-skinflap chamber that allowed for the application of fluorescent microscopy to study probe leakage rates (6, 7). In one model, a human colon cancer xenograft implanted into this dorsal chamber in immuno-deficient mice, it was reported that variation of molecular probe size by a factor of approximately 2 resulted only in a variation of permeability by about the same factor of 2 (6). Such a small factor results from the process of diffusion and its size dependence and not on any difference in pore number for the smaller size probes. This suggests that in these studies the tumor leakiness is dominated by large pores. In the large pore limit, differences in leak rates would be governed by differences in diffusion rates between particle agents. In this and a subsequent study, the large pore cutoff sizes were reported to be as large as 700nm (7).

These results do not agree with a variety of MRI observations for a variety of tumor xenografts in mice and rats and in chemically induced tumors in rats. Thus, there is a need for an explicit determination of tumor endothelial pore size distributions with methods other than used with the dorsal-skin-flap chamber system. The experimental design implemented in this study is straightforward. Some five agents of globular structure ranging in size from approximately 2 to 7nm in radius were compared in rat mammary carcinoma models and DCE MRI was used to determine the probe permeability. A simple model is invoked to relate permeability dependence on probe size to endothelial pore size distribution.

## Materials and Methods

### Permeability measurements

The transport rate of a probe from the intravascular space to the tumor interstitium is at the center of these studies. A two compartment model has been used to describe the transfer of probes from the vascular to the extravascular space:

The quantitative expression for the observed signals in a two compartment model (8, 9) is derived from the expression for variation of agent concentration in the tumor:

(1) $$\frac{\partial C_{T}}{\partial t}=k_{1}\;C_{0}e^{-\gamma t}-k_{2}\;C_{T}$$

where *C_0_* and *C_T_* are the intravascular and the tumor extravascular concentration respectively, and *k_1_* is the input transport rate into the tumor, in inverse time units, min^−1^ and *k_2_* is the rate of leaving the tumor, and γ is the clearance rate from the blood also in min^−1^. The initial slope gives a good approximation of the input rate constant *k_1_* since the second term is small for low tumor concentrations. This is a matter of the kinetic rates in an actual experiment. Alternatively Eqn. 1 can be solved numerically to fit the observed data and *k_1_* can be derived explicitly even if the nonlinearity of the second term is substantially visible in the kinetic data.

The parameter of interest, permeability, or more precisely the permeability-surface area product, PS, is just the *k_1_* rate constant put into appropriate units (μmoles/min*gm). Thus the usual parameter in dynamic contrast enhancement experiments, the PS term, the permeability-surface area product, is just

$$\text{PS}=k_{1}\times 1000\;\mu \text{moles}/\text{min}*\text{gm}$$

when *C_0_* is in μmoles/ml and *k_1_* is deduced from the rate of change of MRI signal in Eqn. 2 below.

The task of measuring *k_1_* is done by MR imaging in a dynamic contrast enhancement method, DCE. The term *C_T_(t)* in Eqn. 1 is related to the observed signal changes by the following relationship:

(2) $$\Delta S_{T}^{f}\;(t)=\frac{S(t)-S_{1}}{S_{0}}=C_{T}\;(t)R_{1}T_{1}$$

The left hand side represents the observed fractional changes in signal intensity for an imaging voxel at time *t* as defined with *S* (*t*) the signal at time *t*, and *S_1_* the signal immediately after injection, i.e. the blood volume signal, and *S_0_* is the signal before contrast agent injection. *C_T_* (*t*) is the concentration of agent in the tumor at time *t*, the extravascular component, *R_1_* is the proton longitudinal relaxivity of the agent, and *T_1_* is the proton relaxation time of the tissue prior to agent injection (usually about 1 sec at a field of 1.5T). The raw experimental data below are expressed as a dimensionless number, per cent enhancement per unit time, the left hand side of Eqn. 2, per unit time, i.e. the observed slope of the fractional signal change of Eqn. 2 in the dynamic enhancement data set. The slope data can be compared directly since all the animal injections were at the same effective dose: actual dose corrected by relaxivity differences between the contrast agents used.

### Dynamic contrast enhancement

A GE Signa 1.5T scanner was used for the animal imaging. The receive coil was a cylindrical solenoid coil, 5 cm diameter, into which the animals were placed under anesthesia (an intraperitoneal injection of a mixture of acepromazine, xylazine, ketamine, and atropine). A T_1_ spin echo sequence was used to generate the images: TE/TR 10/250 msec, Nex 2, 12 × 12 FOV, 1mm slice thickness. An image was obtained prior to agent injection. At t = 0 the contrast polymeric agent was injected by tail vein at dose of 0.025mmole Gd/kg. The first image was obtained at t = 1 minute and subsequent images were obtained every 4 minutes for up to 40 minutes post injection.

To quantify tumor permeability, regions of interest, ROI regions encompassing the whole tumor were taken for 3 to 4 image slices of the tumor. A signal enhancement curve was obtained for each image slice as a function of time using a histogram method for the signal change values for each pixel. Since the tumor permeability responses were heterogeneous as is well known in the literature, we used the highest 10% of the responding pixels to define the angiogenically active tumor permeability areas (2). Inclusion of all the pixels in a tumor slice results in a dilution of the average signal changes in such a slice by a large number of unresponding pixels, associated with presumably necrotic regions of the tumor. In previous reports, in order to avoid signal dilution and focus on the active tumor regions, the analysis regions were operator defined and usually encompassed the tumor rim (3,10–12). Unless otherwise specified the data below for tumor permeability are for the highest 10% of responding tumor pixels. Usually the high responding regions were in the periphery of the tumor as noted previously.

### Permeability and pore sizes

The transport rate term *k_1_* depends on pore size and pore number density in the following way for two cases. 1) Case of diffusion across capillary: The rate of transport across a pore (see for example ref. 13) is inversely proportional to the friction coefficient in the Einstein diffusion equation, D = kT/ *f*, and the friction coefficient is proportional to the hydrodynamic radius of the particle probe, the so called stokes radius. Therefore the transport rate term *k_1_* in Eqn. 1 is given by

(3) $$k_{1}\propto \frac{N_{p}}{f}$$

where *N_p_* is the number of available pores for the probe in question. The transport rate must depend on all the pores that the probe can interrogate, i.e. all pores larger than the size of the particle probe. We can express this dependence in the following way:

(4) $$N_{p}=\int_{\infty }^{R_{H}}\;\eta_{p}(r)dr$$

Here *η_p_*(*r*) is the pore size distribution.

We now make a simple assumption that the pore size distribution is a power law distribution, i.e. *η_p_*(*r*) = *r*^−^*^β^*. This is a natural assumption as many phenomena in nature are described by such power law distributions and are connected to the fractal geometries often found in nature(see for example ref. 14). Furthermore, this assumption will be validated by the data itself and its agreement to a power law dependence. Integration of Eqn. 4 with *η_p_*(*r*) = *r*^−^*^β^* gives

$$N_{p}=r_{H}^{-\beta +1}$$

and Eqn. 3 becomes just

(5) $$k_{1}\propto r_{H}^{-\beta }$$

The transport rate power law exponent is the exponent of the pore size distribution.

Case 2) is convection as the driving force for transport. For this situation the transport rate can be written as

(6) $$k_{1}\propto N_{p}\times \Phi$$

where the term, Φ, encompases hydrostatic pressure and other contributing terms to the fluid flow through a set of pores. Because of convective flow, we have no explicit dependence on particle friction coefficient. And if we keep Φ constant in the experimental design (same conditions for the animals, same anesthesia, same dosages etc.) variation in the observed transport rate *k_1_* as a function of probe size can again be interpreted in terms of pore size distribution by repeating the integration steps shown above. In this case if the observed power law of the transport rate *k_1_* has an exponent −β, then the pore distribution exponent would be (−β +1). In this case, variations in the Φ term would constitute the leading sources of variance in the transport measurements.

As will be shown below in describing the particle probes used, diffusion across small distances such as across a cell gap junction is very fast and diffusion in the MR measurement interval of 4 minutes is large enough to encompass the mean capillary spacing in angiogenic regions of the tumor. Therefore, the diffusion model is clearly applicable to these probes and Eqn. 5 for the pore distribution is a valid interpretation of the experimental observations shown in Figure 2.

### Contrast agent probes

1) Synthesis of Gd-DTPA-polylysine in globular collapsed state.

Polylysine was purchased from Sigma-Aldrich (degree of polymerization by viscosity was 455 and 613 for two different lots used). A mixed anhydride method was used to couple, diethylenetriaminepentaacetic acid, DTPA (Sigma-Aldrich, 98% pure) to the polylysine with modifications from the published protocol (15). DTPA (7 equivalents to 1 of lysine) was placed in dry acetonitrile and tryethylamine (5 equivalents to 1 of DTPA) was added and warmed with stirring until the solution was clear. It was then placed under a N_2_ atmosphere in a −30 °C bath. Isobutylchloroformate, IBCF, was then added slowly and the solution was allowed to mix for 30 minutes. The resulting mixture was then added drop-wise to a solution of polylysine (in 100mM sodium bicarbonate) in an ice bath with stirring over 5 minutes. The solution was allowed to come to room temperature and was stirred for 16 hours. The solution was then placed in a rotary evaporator to eliminate the volatile organic components and then further purified through diafiltration (Amicon 8400 stirred filtration cell, 30,000 Mw cutoff filters). The purified product was then labeled with Gd through addition of Gd-Na citrate until no free Gd was detected in a colorimetric assay (10 μL of solution added to 1 mL of 1μM of Arsenazo III). Free lysines were determined with a trinitrobenzensulfonic acid, TNBS, colorimetric assay (16) calibrated with monomeric lysine, lysine containing peptides, and N-acetyl-lysine. This synthesis protocol resulted in variable conjugation levels of DTPA for different synthesis runs, ranging from 60% to 95%. Increasing the temperature of the ICBF bath to −20 °C and running the coupling reaction for a shorter period, 6 hours, resulted in even lower conjugation levels. These products were later characterized with relaxivity measurements after Gd labeling. Gd content in all the labeled products was determined by ICP-AES.

For purposes of these experiments, only two of the lower conjugation constructs were used that were verified to have a collapsed conformation by size exclusion chromatography and dynamic light scattering measurements of hydrodynamic size. For more details on Gd-DTPA-polylysine conformation see previously publisher reports (17,18).

2) Synthesis of Gd-DTPA-albumin and Gd-DTPA-IgG

An identical protocol was followed as above. In the coupling reaction, the mixed anhydride slurry was added to a solution of either bovine serum albumin or IgG (Sigma-Aldrich, 100mg in 12 mL 100 mM sodium bicarbonate). Coupling proceeded for 16 hours and then the same purification procedures were undertaken as for the polylysine. Gd content was determined by ICP-AES.

3) Use of Gad 17 data relative to Gd-DTPA-albumin

Gad 17 is a dendrimeric polymer agent whose hydrodynamic size (mean of two values given in the literature, radius of 2 nm +/−0.2 nm) extends the size range of our present investigation (limited from 3.5nm for albumin to 6.5nm for the IgG constructs) to allow a more meaningful power law range fit. A detailed study on the kinetics of Gad 17 and Gd-albumin in a rat mammary adenocarcinoma model was reported (10). The permeability of Gad 17 was found to be 12 times higher than for Gd-albumin for the tumor periphery, corresponding to the high 10% of responding pixels: PS = 10.3 μl/min*cc and 0.83 μl/min*cc respectively. I use this multiplying factor of 12 for our observed Gd-albumin slope to fold the Gad 17 permeability into our data set of uptake slope vs. probe hydrodynamic radius. I note that the baseline Gd-albumin permeability values for the two rat mammary adenocarcinoma models were similar, 0.83 vs. 1.6 μl/min*cc, and no further adjustments in projected uptake slope for a Gad 17 size probe was made.

### Hydrodynamic sizes

The hydrodynamic size of the constructs used was determined by dynamic light scattering methods. The value for Gd-DTPA-albumin matched literature values and was found to be 3.5 +/−0.2 nm.

### Probe diffusion

From the hydrodynamic sizes it is straightforward to calculate the diffusion coefficients of these probes given the well determined value for albumin. We find then, that for water and 37 °C, that the diffusion coefficients are: 1.4 × 10^−6^ for Gad 17, the smallest probe, 8.1 × 10^−7^, for albumin, and 4.4 × 10^−7^ for IgG the largest(in units of cm^2^/sec). To cross a cell gap junction of say 2 μm, the time required using the standard diffusion equation, *x^2^* = *2Dt,* is 0.014, 0.024, and 0.045sec respectively. And in a MR measurement time interval of 4 minutes, the diffusion distances are 259, 197, and 145 μm respectively. These distances easily encompass the mean capillary spacing of angiogenic tissue, some 100 μm, (from a blood volume fraction of 4% and mean capillary diameter of 20 μm).

### Polymer conformation

The two Gd-DTPA-polylysine polymers used in these experiments were analyzed for polymer conformation by size exclusion chromatography as previously described (21,22). The two constructs in the present experiments eluted at the times predicted for a collapsed globular conformation matching the behavior of protein standards in the same molecular size range. A coiled polymer in a collapsed globular conformation elutes at a much later time for the same monomer number than one in an extended conformation. It was important that only globular constructs be used for the pore distribution experiments because the extended- linear polymer constructs are known to translocate through the tumor endothelium by a different process, that of polymer reptation (17, 19), than the particle-like probes which extravasate by diffusion/convection through available openings in the endothelium.

### Animal model

A rat mammary adenocarcinoma cell line, Mat B3-13762, ATCC – CRL 1666, was used to implant into female Fisher 344 rats. Subcutaneous tumors were grown by implanting 2 × 10^6^ cells in 0.2ml Hank’s balanced salt solution beneath the dorsal skin flap while the animal was anesthesized. After about 8–10 days the tumors reached a size of between 5 and 10 mm in diameter and the imaging experiments then commenced. Tumors larger than about 15mm in diameter were not used in these studies as the presence of extensive necrotic regions complicated the goal of assessing the active angiogenic tumor permeability and tumor blood volume in the viable regions of the tumor.

All animal experiments were conducted in accordance with the approval of Institutional Animal Care and Use Committee and followed the New York State Health Department regulations.

### Proton relaxivity

The longitudinal proton relaxivity of the various molecular constructs was measured at a field of 1.5T and 25 °C by standard methods (12).

## Results and Discussion

For proper normalization of dose, so that the observed signal slopes could be directly compared between agents used, the R_1_ values of the constructs were measured and the doses were adjusted relative to Gd-DTPA-albumin construct (R_1_ = 15 +/−0.5 mM^−1^ sec^−1^). The relaxivity of the two collapsed polymer constructs were 11.5 and 10.5 mM^−1^ sec^−1^ with 40 and 20% free lysines respectively. The relaxivity of the IgG construct was 15.5 mM^−1^ sec^−1^.

The hydrodynamic radii of the albumin and the IgG constructs were 3.5 +/−0.2 nm and 6.5 +/−1.0 nm, and the two collapsed polylysine constructs were 3.7 +/−0.3 and 5.3 +/−0.3 nm in hydrodynamic radius. The differences in polylysine sizes was driven largely by degree of intramolecular ionic bonding—the construct with the higher free lysine content had a smaller size as expected from molecular modeling calculations (17).

In a related study of breast cancer hemodynamics, DCE data were taken on a xenograft of human breast cancer, MCF7, in immunodeficient mice. This data for Gd-DTPA-albumin probes for MCF-7 could be compared directly with the literature (2). The vascular volume fraction and the PS permeability values for the highest 10% of responding pixels were found to be 31 +/−3 μl/gm and 0.9 +/−0.4 μl/min*gm and compare to 33 +/−5 μl/gm and 1.5 +/−0.3 μl/min*gm found in the Bhujwalla laboratory (2). The results are in agreement for the vascular volume fraction but differ somewhat for the permeability. This difference could be due to the sensitivity of transport to experimental factors and errors or to orthotopic (Bhujwalla laboratory) vs. subcutaneous (this work) implantation of tumors. The former may be more likely as differences of factor of 2 in permeability were observed for subcutaneous Mat lylu tumors (results from this laboratory compared to results from ref. 2, also subcutaneous) and as mentioned above differences for rat mammary adenocarcinoma between ref. 10 and results of this laboratory. However, such variation does not materially effect the conclusions regarding transport rate power law steepness shown in Figure 2 as those rate changes encompass changes of several orders of magnitude.

**Figure 1.**
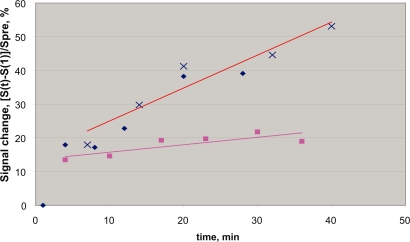
DCE data for two contrast agents Gd-albumin(upper data set) and Gd-IgG (lower data set) for identical effective dose of 0.025 mmole Gd/kG. The fractional signal change is as defined in Eqn. 2 and is for the highest 10% of the responding image pixels. The slope of this fractional change is indicated by the solid lines and is proportional to the uptake rate of the agent into the tumor, i.e., the PS, permeability-surface area product for the agent.

Raw DCE data are shown in Figure 1 for albumin and IgG probes for the rat mammary adenocarcinoma model. There is a large difference in signal slope for the two probes of 3.5 and 6.5 nm hydrodynamic radii respectively.

**Figure 2.**
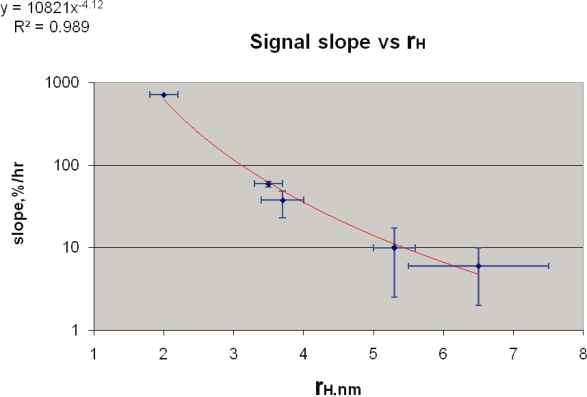
Fractional signal slope, as in Figure 1, plotted as function of contrast agent hydrodynamic radius for rat mammary adenocarcinoma tumors. Data points on right, this work(The number of animals for each experimental point form left to right, are 7, 3, 3, and 3). Upper left data point, extrapolated to present slope values from PS values given relative to albumin (ref. 10) as detailed in text. Power law fit over this range of sizes gives an exponent of −4.1 with R2 of 0.989.

All of the slope data, including the extrapolated value for the 2 nm radius probe that is derived from an earlier study on a rat adenocarcinoma model, are shown in Fig. 2. The data show a very steep drop in the transport rate, i.e., the initial slope of the DCE signals vs. time, as a function of probe size. The power law fit has an exponent of −4.1 with a R^2^ value of 0.989. This exponent of −4.1 +/−0.3 is therefore the exponent of the pore size distribution for diffusion transport. The power law is tested over the range of sizes from 2 to 6.5 nm in radius and over this range, the transport rate is seen to vary by two orders of magnitude, a steep dependence indeed.

### Comparisons with other MRI observations

Confirming a steep pore distribution was the study of Walker 256 sarcoma in rats (4) in which there was a 13 fold change in uptake rate when Gad 17, a 30kD dendrimer construct (2nm radius) was compared to Gd-DTPA-polylysine (a construct of polymerization number 70, 50kD, and a computed radius of ∼3.4 nm radius from measurements of Gd-polylysine constructs of various degrees of polymerization, form 100 to 800, and the corresponding dynamic light scattering measurements of hydrodynamic radius, data not shown). An even larger difference in uptake slope between these agents, >13, was observed for rat mammary adenocarcinoma R3230. Thus probe size changes of about a factor of 1.7 results in a change of uptake rate by a factor of 13 and even greater than 13 in the two rat tumor models (consistent with a power law exponent for transport rate of −4.8.

I have already remarked on the comparison of Gd-albumin with Gad 17 in a rat mammary adenocarcinoma model (10). A factor of 12 in the PS value was observed between Gad 17 (2 nm) and Gd-albumin (3.5 nm) which is consistent with a power law exponent of −4.5.

Yet another example of a steep dependence on probe size can be found in the study of chemically induced tumors in rats (11). The permeability of the induced malignant tumors showed a steep dependence on probe size when an albumin agent was compared to a rapid clearance 6.7kD agent P792—a construct that has a disc structure of approximately 3nm radius and ∼ 1.2 nm half width (20).

Interestingly in this study, the benign tumors were not leaky to the albumin agent, PS = 0 +/−0.01 μl/min*gm, whereas the smaller P792 construct showed no significant difference in PS values between malignant and benign tumors. The role of pore size distribution in benign vs. malignant may therefore be an interesting question of tumor biology and also an important factor for discriminating between the tumor types.

### Connection to other tumor models

How general is this rule for pore sizes? Using this pore size distribution model we can go further and compare to several very different studies involving relatively large probes. In one, iron oxide particles, Clariscan particles, of ∼5.5 to 6 nm radius, were used in DCE measurements of human breast tumors (21). The DCE curve, enhancement vs. time, given for an active tumor, could be used to deduce *k_1_* quantitatively and the value is within a factor of 2 from the projection of *k_1_* from the scaling behavior shown in Figure 2 for rat mammary adenocarcinoma tumor models.

In another study, a MPEG agent, a large polymer construct, was used in a rat mammary carcinoma model and its uptake rate into tumors was determined by radiolabeling and tumor concentration measurements at a number of time points over 24 hours (22). The deduced *k_1_* rate from the reported agent concentration data and the projected rate for this agent (given its size from molecular weight determinations) are within a factor of 2 of each other.

Also, using the data for radiolabeled MION -Tf particles and the tissue activity for tumors with transferrin receptors after a fixed period (23), it is possible to estimate the *k_1_* rate given the blood circulation time and the size of the particles. And if the size of these particles is as stated, i.e. to be equivalent to 770kD proteins (24), than the *k_1_* rate that is projected from the power law scaling agrees with the observed tissue values in this experiment.

We see then, that for a variety of tumors, including human breast cancer and for varying particle formulations, and with MR imaging as well as with radiolabel methods, the steep pore size scaling demonstrated in Figure 2, holds true in a general way. Transport rate projections through the use of this quantitative model and these other observations of tumor uptake agree in one case and differ by a factor of 2 in other cases—within errors of the model prediction as shown in the semilog plot of Figure 2 (note that a factor of 2 is 0.3 units of the log scale). This agreement suggests that even for differing experimental conditions and tumor types the general trend of Figure 2 seems to hold.

## Conclusions

A steep power law, $k_{1}\propto r_{H}^{-\beta }$, for the forward transendothelial transport rate as function of probe size was observed for a rat mammary adenocarcinoma model. As a consequence, the tumor endothelial pore size distribution has a steep dependence also with an exponent of −4.1 for transport by diffusion (and −3.1 for transport by convection alone). This steep distribution appears to hold for a number of other tumor models and includes chemically induced tumors in rats. Further reports in the literature on yet other tumor models appear also to be consistent with this quantitative power law dependence of transport rate on probe size and include comparisons to human breast cancer uptake rates with small iron oxide particle agents.

There was no evidence for large pore dominance in any of these tumor models. From reports using two different size contrast agents, there is evidence that benign and malignant tumors may have different endothelial pore size distributions. Present clinical contrast agents, being of small molecular size, may not be ideal for capturing these differences and a better understanding of tumor endothelial pore size distributions may lead to improved contrast agents or more effective therapeutic constructs.

## References

[1] Folkman J (1992). The Role of Angiogenesis in Tumor Growth. Semin. Cancer Res.

[2] Bhujwalla Z, Artemov D, Natarajau K, Ackerstaff E, Solalyappan M (2001). Vascular Differences Detected by MRI for Metastasic Versus Nonmetastatic Breast and Prostate Xenografts. Neoplasia.

[3] Daldrup H, Shames DM, Wendland M, Okuhata Y, Link TM, Rosenau W, Lu Y, Brasch RC (1998). Correlation of Dynamic Contrast –enhanced MR. Imaging with Histologic Tumor Grade: Comparison of Macromolecular and Small-molecular Contrast Media. Am. J. Roentgenol.

[4] Su MY, Muhler A, Lao X, Nalcioglu O (1998). Tumor Characterization with Dynamic Contrast Enhancement MRI Using MR Contrast Agents of Various Molecular Weights. Mag. Reson. Med.

[5] Su MY, Wang Z, Carpenter PM, Lao X, Muhler A, Nalcioglu O (1999). Characterization of N-ethyl-N-Nitrosourea-Induced Malignant and Benign Breast Tumors in Rats by Using Three MR Contrast Agents. J. Magn. Reson. Imag.

[6] Yuan F, Dellian M, Fukumura D, Leunig M, Berk DA, Torchilin VP, Jain RK (1995). Vascular Permeability in a Human Tumor Xenograft: Molecular Size Dependence and Cutoff Size. Cancer Res.

[7] Hobbs SK, Monsky WL, Yuan F, Roberts WG, Griffith L, Torchilin VP, Jain RK (1998). Regulation of Transport Pathways in Tumor Vessels: Role of Tumor Type and Microenvironment. Proc. Natl. Acad. Sci., U.S.A.

[8] Tofts PS, Kermode AG (1991). Measurement of the Blood-brain Barrier Permeability and Leakage Space using Dynamic MR Imaging. Fundamental Concepts. Magn. Reson. Med.

[9] Su MY, Jao JC, Nalcioglu O (1994). Measurement of Vascular Volume Fraction and Blood-tissue permeability Constants with Pharmacokinetic Model: Studies in Rat Muscle Tumor. Magn. Reson. Med.

[10] Demsar F Shames DM Roberts TPL Stiskal M Roberts HC Brasch RC 1998 Kinetics of MRI Contrast Agents with a Size Ranging Between Gd-DTPA and Albumin-Gd-DTPA: Use of Cascade-Gd-DTPA-24 Polymer Electro and Magnetobiology 17 283 97+

[11] Turetschek K, Floyd E, Shames DM, Roberts TPL, Preda A, Novikov V, Corot C, Carter WO, Brasch RC (2001). Assessment of a Rapid Clearance Blood Pool MR. Contrast Medium (P792) for Assays of Microvascular Characteristics in Experimental Breast Tumors With Correlations to Histopathology. Mag. Reson. Med.

[12] Uzgiris EE (2004). The Role of Molecular Conformation on Tumor Uptake of Polymeric Contrast Agents. Inv. Radiology.

[13] Deen WM, Bohrer MP, Epstein NB (1981). Effects of Molecular Size and Conformation on Diffusion in a Microporous Membrane. AIChE Journal.

[14] Feder J (1983).

[15] Sieving PF, Watson AD, Rocklage M (1990). Preparation and Characterization of Paramagnetic Polychelates and Their Protein Conjugates. Bioconjugate Chem.

[16] Fields R (1972). The Rapid Determination of Amino Groups with TNBS. Methods in Enzymology.

[17] Uzgiris EE, Cline H, Moasser B, Grimmond B, Amaratunga M, Smith JF, Goddard G (2004). Conformation and Structure of Polymeric Contrast Agents for Medical Imaging. Biomacromolecules.

[18] Uzgiris EE, Sood A, Bove K, Grimmond B, Lee D, Lomnes S (2006). A Multimodal Contrast Agent for Preoperative MR Imaging and Intraoperative Tumor Margin Delineation. Technology in Cancer Treatment and Research.

[19] Uzgiris EE (2008). A Cell- Surface Polymer Reptation Mechanism for Tumor Transendothelial Transport of Macromolecules. Technology in Cancer Treatment and Research.

[20] Port M, Corot C, Rousseaux O, Raynal I, Devoldere L, Idee J-M, Dencausse A, Greneur SL, Simonot C, Meyer D (2001). P792: A Rapid Clearance Blood Pool Agent for Magnetic Resonance Imaging: Preliminary Results. Mag. Res. Materials in Physics, Biology, and Medicine.

[21] Rydland J, Bjornerud A, Haugen OA, Torheim G, Torres C, Kvistad KA, Haraldseth O (2003). New Intravascular Contrast Agent Applied to Dynamic Contrast Enhanced MR Imaging of Human Breast Cancer. Acta Radiologica.

[22] Bogdanov A, Wright SC, Marecos EM, Bogdanova A, Martin C, Petherick P, Weissleder R (1997). A Long-circulating Co-polymer in “Passive Targeting” to Solid Tumors. J. Drug Targeting.

[23] Weissleder R, Moore A, Mahmood U, Bhorade R, Benvenviste H, Chiocca EA, Basilion JP (2000). In vivo Magnetic Resonance Imaging of Transgene Expression. Nature Med.

[24] Moore A, Basilion JP, Chiocca EA, Weissleder R (1998). Measuring Transferrin Receptor Gene Expression by NMR Imaging. Biochimica et Biophysica Acta.

